# MSGSO: A Multi-Strategy Glider Snake Optimizer for Global Optimization and 3D UAV Path Planning

**DOI:** 10.3390/biomimetics11090616

**Published:** 2026-09-01

**Authors:** Burak Aggul, Amir Seyyedabbasi

**Affiliations:** 1 Department of Database, Network Design and Management, Plato Vocational School, Istanbul Topkapi University, Istanbul 34020, Turkey; 2 Department of Computer Engineering, Faculty of Engineering and Natural Science, Istinye University, Istanbul 34396, Turkey; amir.seyyedabbasi@istinye.edu.tr

**Keywords:** Glider Snake Optimizer, nonlinear dynamic polynomial mutation, elite opposition-based learning, quadratic interpolation, CEC 2019, CEC 2022, ablation study, UAV path planning

## Abstract

Information in the Glider Snake Optimizer (GSO) propagates through a leader–predecessor chain. This structure is simple, but it can lose diversity when adjacent agents converge to the same region. The proposed Multi-Strategy Glider Snake Optimizer (MSGSO) retains the original GSO update and subsequently applies nonlinear dynamic polynomial mutation (NDM), elite opposition-based learning (EOBL), and quadratic interpolation (QI). MSGSO was evaluated on CEC 2019 and CEC 2022 with 30 matched random seeds and 15,000 objective evaluations for every algorithm–problem pair. The experiments included seven alternative optimizers and all single, pairwise, and three-operator GSO variants. Across the 34 benchmark problems, MSGSO significantly outperformed GSO on 31 and showed no significant loss. It nevertheless ranked third in each external comparison: L-SHADE led CEC 2019 and CEC 2022 at D=10, while CMA-ES led CEC 2022 at D=20. The ablation attributed most of the gain to NDM; NDM–GSO led the GSO variants on CEC 2019, and NDM–QI–GSO led at both CEC 2022 dimensions. In the UAV study, MSGSO returned 29 feasible paths in 30 runs in Scenario 1 and feasible paths in every run in the other two scenarios. It led the feasibility-first ranking in Scenarios 1 and 2 and placed third in Scenario 3. Thus, MSGSO improves its parent algorithm, although the full three-operator sequence is not consistently the best configuration.

## 1. Introduction

Metaheuristics are often used when derivatives are unavailable, objective functions are discontinuous or expensive, or feasibility depends on several interacting constraints. Their practical reach extends from continuous engineering design to routing and control, yet parameter sensitivity, scalability, premature convergence, and reproducibility remain persistent concerns [1,2]. The No Free Lunch theorem rules out universal superiority across problem classes [3]. Likewise, a new biological metaphor does not by itself constitute a new search principle [4]. An optimizer should be judged by its mathematical operators and by experiments that separate algorithmic gains from differences in computational effort.

That separation is particularly important for population-based methods. Apparent improvements may reflect a useful search mechanism, but they may also come from extra candidate evaluations, favorable parameter choices, or a restricted benchmark set. Repeated runs, adequate sample sizes, common stopping criteria, problem-level rankings, and paired nonparametric tests are recommended for stochastic optimization studies [5,6,7]. The experiments reported here follow these principles: benchmark comparisons use matched seeds and identical objective-evaluation budgets, CEC 2022 is examined at both supported dimensions, and numerical optimizers are included alongside population-based methods. In the constrained UAV study, feasibility is evaluated separately from path cost.

The Glider Snake Optimizer (GSO) arranges its population as a chain. The best agent guides the search, and every subsequent agent also follows its immediate predecessor [8]. This update is simple to implement, but the chain may lose diversity when the leader and neighboring agents gather in the same region. Their guidance directions then become similar, leaving later agents with little new information. The original formulation has no explicit opposition step, no polynomial mutation schedule, and no interpolation model built from elite solutions. This study investigates whether these mechanisms can strengthen GSO without replacing its defining chain update.

Combining operators with different search roles is well established in hybrid metaheuristics [9]. Recent studies have also shown that multi-strategy modifications can improve the search behavior of different metaheuristic frameworks. For example, multi-strategy mechanisms have been investigated for snow ablation optimization [10] and artificial rabbits optimization [11]. Multi-strategy enhancement of the Secretary Bird Optimization Algorithm has also been investigated for engineering optimization [12]. Our previous work further examined a multi-strategy SBOA for global optimization and UAV path planning [13].

Such combinations can be useful, but their interpretation requires ablation. A final rank alone cannot show which operator produced the gain or whether the gain arose simply because the hybrid evaluated more candidates.

Our previous multi-strategy studies similarly showed that the benefit of an enhanced optimizer depends not only on the inclusion of additional operators, but also on their functional complementarity and interaction within the parent search mechanism. The present study follows this principle by retaining the defining GSO chain update and introducing NDM, EOBL, and QI as complementary variation, diversification, and refinement mechanisms rather than replacing the original search structure. Complete-subset ablation is therefore used to determine whether the observed improvement arises from the full combination or from particular operators and interactions among them.

MSGSO combines three established mechanisms with complementary roles. Nonlinear dynamic polynomial mutation (NDM) perturbs coordinates with a probability that decreases during the search [14]. Opposition-based learning evaluates a point together with an opposite point [15]; elite opposition-based learning (EOBL) adapts this idea to population extrema and elite guidance [16]. Quadratic interpolation (QI) uses the objective values of selected solutions to estimate a coordinate-wise local minimizer [17,18]. In the proposed sequence, NDM restores local variation, EOBL samples population-relative opposite regions, and QI refines the search near the current elite set. The experimental study examines these effects individually and in every possible operator subset.

The numerical benchmarks are complemented by a three-dimensional UAV path planning problem. Improved metaheuristic methods have been applied to UAV path planning in complex environments [19]. Multi-strategy pelican optimization has also been investigated for UAV path planning [20]. More recent work has considered multiobjective formulations with adaptive perturbation and opposition-based search [21].

The present study keeps the MSGSO benchmark configuration unchanged and tests it in three static, single-objective environments. Dynamic obstacles, multiobjective decision making, and flight-level validation remain outside its scope.

The main contributions of this paper can be summarized as follows:

- A sequential MSGSO architecture integrates NDM, EOBL, and QI after the original GSO update. The role and objective-evaluation cost of each stage are specified explicitly.
- An evaluation-matched study compares MSGSO with seven optimizers on CEC 2019 and on CEC 2022 at D=10 and D=20. Complete-subset ablation and one-at-a-time parameter variation analyses identify the contribution of each operator and the dependence on parameter settings and operator order.
- A 500-iteration UAV study evaluates feasibility, conditional path cost, constraint violations, and feasibility-first rankings in three static three-dimensional environments.

Section 2 reviews GSO. Section 3 defines the three strategies and their integration. Section 4 presents the experimental results and analysis. Section 5 reports the UAV study. Section 6 concludes the paper and future work.

## 2. Glider Snake Optimizer

GSO maintains a population of *N* agents in a chain. The leading agent is the best solution found in the current population; each remaining agent is guided by both this leader and the agent immediately ahead of it [8]. Let *D* be the problem dimension, *t* the current iteration, and *T* the maximum number of iterations. The position of agent *i* is $S_{i}\left(t\right)\in \left[\mathrm{lb},\mathrm{ub}\right]$. Initial positions are sampled uniformly as

(1) $$S_{i}\left(0\right)=\mathrm{lb}+r_{i}⊙\left(\mathrm{ub}-\mathrm{lb}\right),\;r_{i}∼U{\left(0,1\right)}^{D}.$$

After evaluation, the agents are sorted in ascending fitness order because all problems considered here are minimization problems. The current leader is $L\left(t\right)=S_{1}\left(t\right)$. For every non-leading agent, the displacement toward the leader and the displacement toward its predecessor are

(2) $$\begin{array}{cc} D_{i}^{CL}\left(t\right) & =L\left(t\right)-S_{i}\left(t\right), \end{array}$$

(3) $$\begin{array}{cc} D_{i}^{PL}\left(t\right) & =S_{i-1}\left(t\right)-S_{i}\left(t\right). \end{array}$$

These two directions are combined in the chain update

(4) $$S_{i}\left(t+1\right)=S_{i}\left(t\right)+A\left(t\right)\left(D_{i}^{CL}\left(t\right)+D_{i}^{PL}\left(t\right)\right),$$

where

(5) $$A\left(t\right)=1-\frac{t}{T}.$$

Thus, the step coefficient decreases linearly from one to zero. Early updates allow relatively broad movement; later updates place progressively more weight on local refinement.

GSO also applies a replacement rule to weak solutions. An agent in the lower half of the sorted population is selected for replacement with probability RP. Following the released implementation, let $r_{1}$, $r_{2}$, and $r_{3}$ be distinct population indices and let $F_{i}$ denote the fitness of agent *i*. The corresponding coordinate update is

(6) $$S_{i,d}\left(t+1\right)=\frac{r_{1}}{N}S_{r_{1},d}\left(t\right)+A\left(t\right)\left(\frac{F_{1}}{F_{i}}+\frac{F_{r_{2}}}{F_{r_{3}}}\right).$$

Here, $F_{1}$ is the leader fitness and $F_{i}$ is the fitness of the weak agent. The experiments use the source value RP=0.50. Any ratio with a zero or non-finite denominator is set to zero. Agents are updated in descending, in-place chain order, then clipped to the search bounds and evaluated. Both this rule and the original chain update are retained in MSGSO.

## 3. Multi-Strategy Glider Snake Optimizer

MSGSO augments the original GSO cycle with three successive stages: NDM for coordinate-level variation, EOBL for population-relative exploration, and QI for local refinement around elite solutions. The following subsections define each stage before describing the complete sequence.

### 3.1. Strategy 1: Nonlinear Dynamic Polynomial Mutation

NDM perturbs individual coordinates, with a mutation probability that decreases nonlinearly as the search progresses [14]:

(7) $$p_{m}\left(t\right)=p_{\max}-\left(p_{\max}-p_{\min}\right){\left(\frac{t}{T}\right)}^{k}.$$

For a selected coordinate, a random value u∼U(0,1) determines the polynomial displacement

(8) $$\delta \left(u\right)=\left\{\begin{array}{cc} {\left(2u\right)}^{1/(\eta_{m}+1)}-1, & u<0.5,\\ 1-{\left[2(1-u)\right]}^{1/(\eta_{m}+1)}, & u\geq 0.5, \end{array}\right.$$

which gives the candidate coordinate

(9) $$S_{i,d}^{\prime }=S_{i,d}+\delta \left(u\right)\left(ub_{d}-lb_{d}\right).$$

The candidate is clipped to the global bounds before evaluation. The distribution index is $\eta_{m}=20$, as in [14]; the remaining parameters are $p_{\max}=0.50$, $p_{\min}=0.01$, and k=2. MSGSO applies NDM to every agent immediately after the GSO update. Each mutated solution is compared with the agent from which it was generated, and only the better of the two survives.

### 3.2. Strategy 2: Elite Opposition-Based Learning

EOBL derives a separate interval for each coordinate from the current population [16]:

(10) $$da_{d}\left(t\right)=\min_{i}S_{i,d}\left(t\right),\;db_{d}\left(t\right)=\max_{i}S_{i,d}\left(t\right).$$

Given $r_{i,d}∼U\left(0,1\right)$, the corresponding opposite coordinate is

(11) $$S_{i,d}^{opp}\left(t\right)=r_{i,d}\left(da_{d}\left(t\right)+db_{d}\left(t\right)\right)-S_{i,d}\left(t\right).$$

An opposite coordinate outside the global search interval is reinitialized by

(12) $$S_{i,d}^{opp}\left(t\right)=lb_{d}+v_{i,d}\left(ub_{d}-lb_{d}\right),\;v_{i,d}∼U\left(0,1\right).$$

MSGSO computes these dynamic bounds from a snapshot of the post-NDM population and generates one opposite candidate for each agent. The candidate is evaluated against its source agent, again using greedy one-to-one selection. Because all bounds come from the same population snapshot, the result does not depend on the order in which agents are processed.

### 3.3. Strategy 3: Quadratic Interpolation

QI fits a quadratic model to three solutions and their objective values, then uses the model to estimate a point near the local minimum [17]. Let $\left(x_{1},f_{1}\right)$, $\left(x_{2},f_{2}\right)$, and $\left(x_{3},f_{3}\right)$ denote three distinct solutions and their corresponding objective values. For each dimension *d*, the QI candidate is calculated as

(13) $$q_{d}=\frac{1}{2}\frac{f_{1}\left(x_{2d}^{2}-x_{3d}^{2}\right)+f_{2}\left(x_{3d}^{2}-x_{1d}^{2}\right)+f_{3}\left(x_{1d}^{2}-x_{2d}^{2}\right)}{f_{1}\left(x_{2d}-x_{3d}\right)+f_{2}\left(x_{3d}-x_{1d}\right)+f_{3}\left(x_{1d}-x_{2d}\right)}.$$

MSGSO applies QI after EOBL, using the three best distinct solutions in the current population. If the denominator in Equation (13) is numerically close to zero, that coordinate is copied from the best of the three solutions. The resulting point is clipped to the search bounds and evaluated. It replaces the worst population member only when it improves the global best.

**Figure 1:**
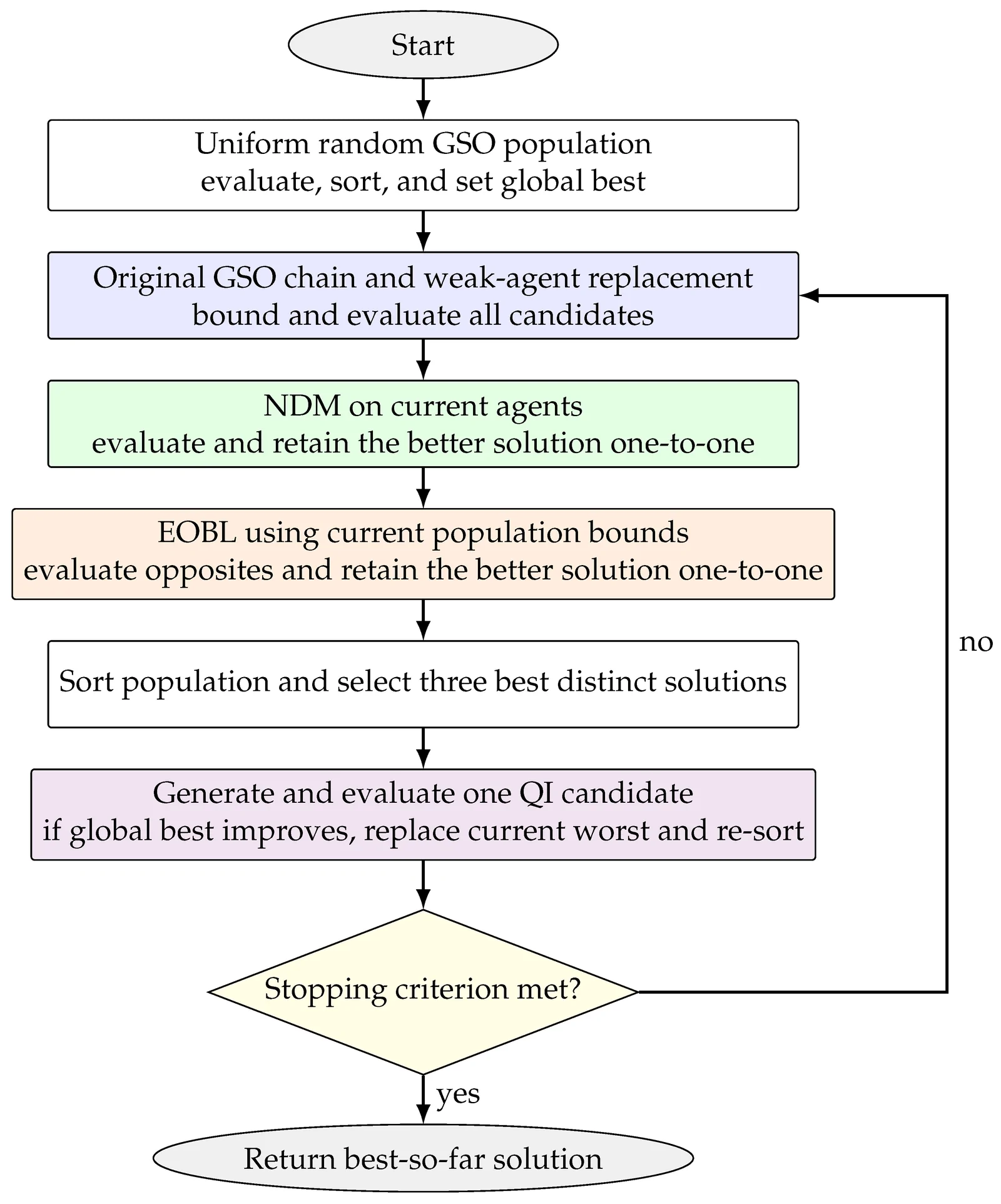
Flowchart of the MSGSO procedure.

The complete cycle begins with the original GSO chain movement and weak-agent replacement. NDM then acts on the updated agents, after which EOBL uses the post-NDM population to construct its dynamic bounds. The population is sorted once more, and its three best distinct members define a single QI candidate. Greedy selection at the NDM and EOBL stages prevents either operator from degrading its source solution; the QI stage is accepted only if it improves the global best. This selection rule preserves the best-so-far solution throughout the run. Figure 1 summarizes this sequence, while Algorithm 1 provides the corresponding pseudocode.
**Algorithm 1** Pseudocode of MSGSO  1: Generate *N* uniform random solutions using Equation (1)  2: Evaluate and sort the population and initialize the best-so-far solution  3: **while** the active stopping criterion has not been met **do**  4:     Increment *t* and update A(t) and $p_{m}\left(t\right)$  5:     Apply the original GSO update using Equations (4) and (6)  6:     Bound and evaluate all GSO candidates  7:     **for** each current agent *i* **do**  8:         Generate an NDM candidate using Equations (7)–(9)  9:         Evaluate the candidate and retain it if it is better than agent *i*10:     **end for**11:     Determine the EOBL bounds using Equation (10)12:     **for** each current agent *i* **do**13:         Generate its opposite solution using Equations (11) and (12)14:         Evaluate the opposite solution and retain the better solution15:     **end for**16:     Sort the population and select the three best distinct solutions17:     Generate one QI candidate using Equation (13)18:     Bound and evaluate the QI candidate19:     **if** the QI candidate improves the global best **then**20:         Replace the worst population member and re-sort21:     **end if**22:     Update the leader and best-so-far solution23:  **end while**24:  **return** Best-so-far fitness and solution

After initialization, a complete MSGSO cycle requires at most N+N+N+1=3N+1 objective evaluations: *N* for GSO, *N* for NDM, *N* for EOBL, and one for QI. The QI evaluation is omitted when three numerically distinct elite solutions are unavailable. In the CEC experiments, the number of nominal cycles is estimated from the active stages and used in the schedules of Equations (5) and (7). Execution stops as soon as the shared evaluation budget is reached, even if the last cycle is incomplete. The UAV study instead records 500 complete iterations together with the exact number of objective evaluations consumed by each method.

## 4. Experimental Results and Analysis

The numerical experiments were organized into four parts: an eight-method comparison, complete-subset ablation, a one-at-a-time parameter variation analysis, and a study of convergence as a function of objective evaluations. CEC 2022 was tested at both supported dimensions.

### 4.1. Experimental Configuration and Benchmark Functions

All benchmark runs were executed in MATLAB R2026a Update 1 on a computer with an Intel Core i9-14900HX processor and 64 GB of RAM. Each algorithm–problem pair was repeated 30 times with seeds 1–30; the same seed was assigned to every algorithm in a matched run. The population size was N=30 unless the method defined its own population schedule, and every implementation clipped candidate solutions to the global bounds.

Every CEC run used exactly 15,000 objective evaluations. At N=30, this is equivalent to 500 full population evaluations. The budget was applied to all 14 algorithmic configurations and all 34 benchmark problems. It is smaller than the official CEC 2022 competition allowance of 200,000 evaluations at D=10 and 1,000,000 at D=20 [22]; the reported values represent performance under the stated common budget, not competition scores.

In total, the benchmark study generated 14,280 run files and 214,200,000 objective evaluations. Every run reached the intended budget. The recorded best-so-far sequence was monotone in every file, and the tables and figures were produced directly from these records.

The CEC 2019 functions were evaluated at their prescribed dimensions and bounds; all have $f^{★}=1$ [23]. CEC 2022 was evaluated at D=10 and D=20 over ${\left[-100,100\right]}^{D}$, using the official shift data, rotations, and biases [22]. The two suites are listed in Table 1 and Table 2.

The external comparison included MSGSO, GSO, GWO [24], WOA [25], PSO [26], DBO [27], CMA-ES [28], and L-SHADE [29]. CMA-ES used Hansen’s reference MATLAB implementation (version 3.61.beta). L-SHADE followed the authors’ MATLAB/Octave release 1.0.1 through a formula-equivalent MATLAB interface. The original CMA-ES core was not modified. Lightweight wrappers handled bounds, evaluation logging, and termination at the shared budget. No comparator was tuned for this benchmark set; published settings or reference code defaults were fixed before the experiments. The MSGSO configuration was also fixed before, and independently of, the subsequent parameter variation analysis. The implementation sources and fixed parameter settings of all compared methods are summarized in Table 3.

Ranking was performed separately for the eight external methods and the eight GSO variants. Within each benchmark function, methods were ranked by the median of 30 runs; equal medians received average ranks in the manner of Friedman [30]. Win/tie/loss (W/T/L) counts were obtained from paired, two-sided Wilcoxon signed-rank tests on matched seeds at α=0.05 [31]. A win indicates a significant advantage for MSGSO, a loss a significant advantage for the comparator, and a tie a non-significant difference. The reported *p*-values are unadjusted.

### 4.2. Complete-Subset Ablation Study

**Figure 2:**
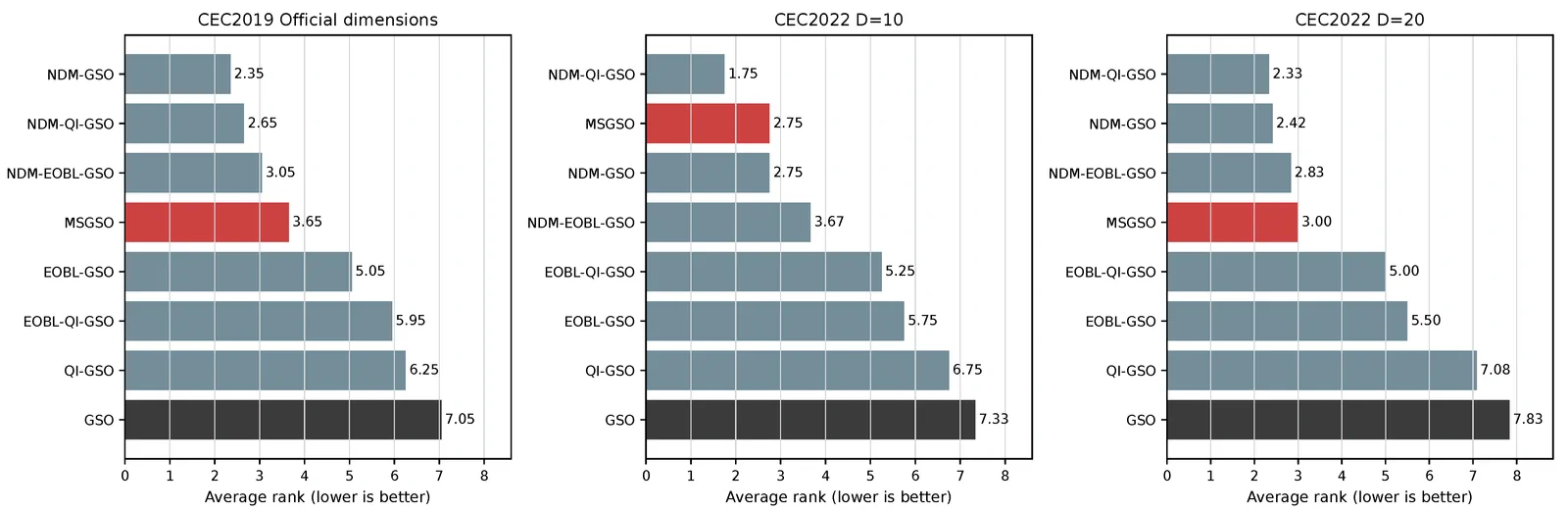
Average ranks in the evaluation-matched complete-subset ablation. Red denotes MSGSO, dark gray denotes the parent GSO, and blue-gray denotes the remaining ablation variants.

The ablation covered the parent GSO, each single-operator extension, all three two-operator combinations, and the complete NDM–EOBL–QI sequence. Every variant received 15,000 evaluations. This design measures the effect of operator choice without giving the multi-operator variants a larger evaluation allowance. Table 4 reports average ranks and W/T/L counts; Figure 2 shows the same rank profiles graphically.

Against the parent GSO, MSGSO recorded 8/2/0 on CEC 2019 and 11/1/0 and 12/0/0 on CEC 2022 at D=10 and D=20, respectively. This pattern is consistent across the suites, but the complete three-operator sequence is not the best GSO variant. NDM–GSO achieved the lowest average rank on CEC 2019 (2.350), while NDM–QI–GSO led both CEC 2022 settings, with ranks of 1.750 at D=10 and 2.333 at D=20. MSGSO’s W/T/L records against NDM–QI–GSO were 0/7/3, 0/10/2, and 1/7/4. Taken together, the ablation assigns the largest share of the improvement to NDM and indicates that QI is useful when paired with it. Adding EOBL to that pair did not yield a consistent further gain under an equal evaluation budget.

### 4.3. Comparative Optimization Performance

**Figure 3:**
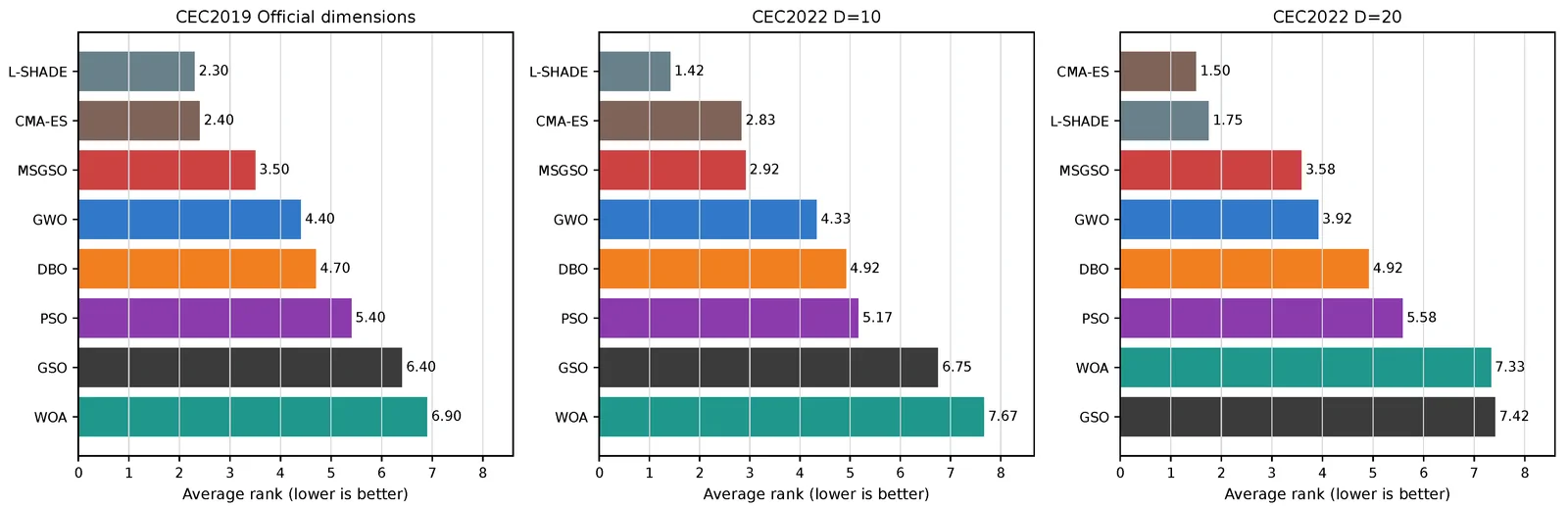
Average ranks in the external eight-method comparison. Each color represents a different algorithm and is used consistently across all three benchmark settings.

Table 5 gives the average ranks and W/T/L counts for the external comparison. Problem-level medians appear in Table 6, Table 7 and Table 8, and Figure 3 provides a graphical summary of the three rankings.

MSGSO placed third in all three settings, but the two leading methods changed with the benchmark. On CEC 2019, L-SHADE and CMA-ES obtained average ranks of 2.300 and 2.400, followed by MSGSO at 3.500. MSGSO outperformed GSO on eight functions, tied on two, and lost on none; against L-SHADE and CMA-ES, its records were 3/0/7 and 2/3/5. At CEC 2022 D=10, L-SHADE led with a rank of 1.417, CMA-ES followed at 2.833, and MSGSO obtained 2.917. Here MSGSO lost all 12 function-level comparisons with L-SHADE and recorded 2/4/6 against CMA-ES. At D=20, CMA-ES ranked first (1.500), followed by L-SHADE (1.750) and MSGSO (3.583). MSGSO again defeated GSO on every function, but recorded only one win against CMA-ES and none against L-SHADE. The proposed method consistently improves GSO and is competitive with the other population-based comparators, while CMA-ES and L-SHADE remain stronger numerical baselines on most of the CEC tests.

### 4.4. One-at-a-Time Parameter Variation Analysis

**Figure 4:**
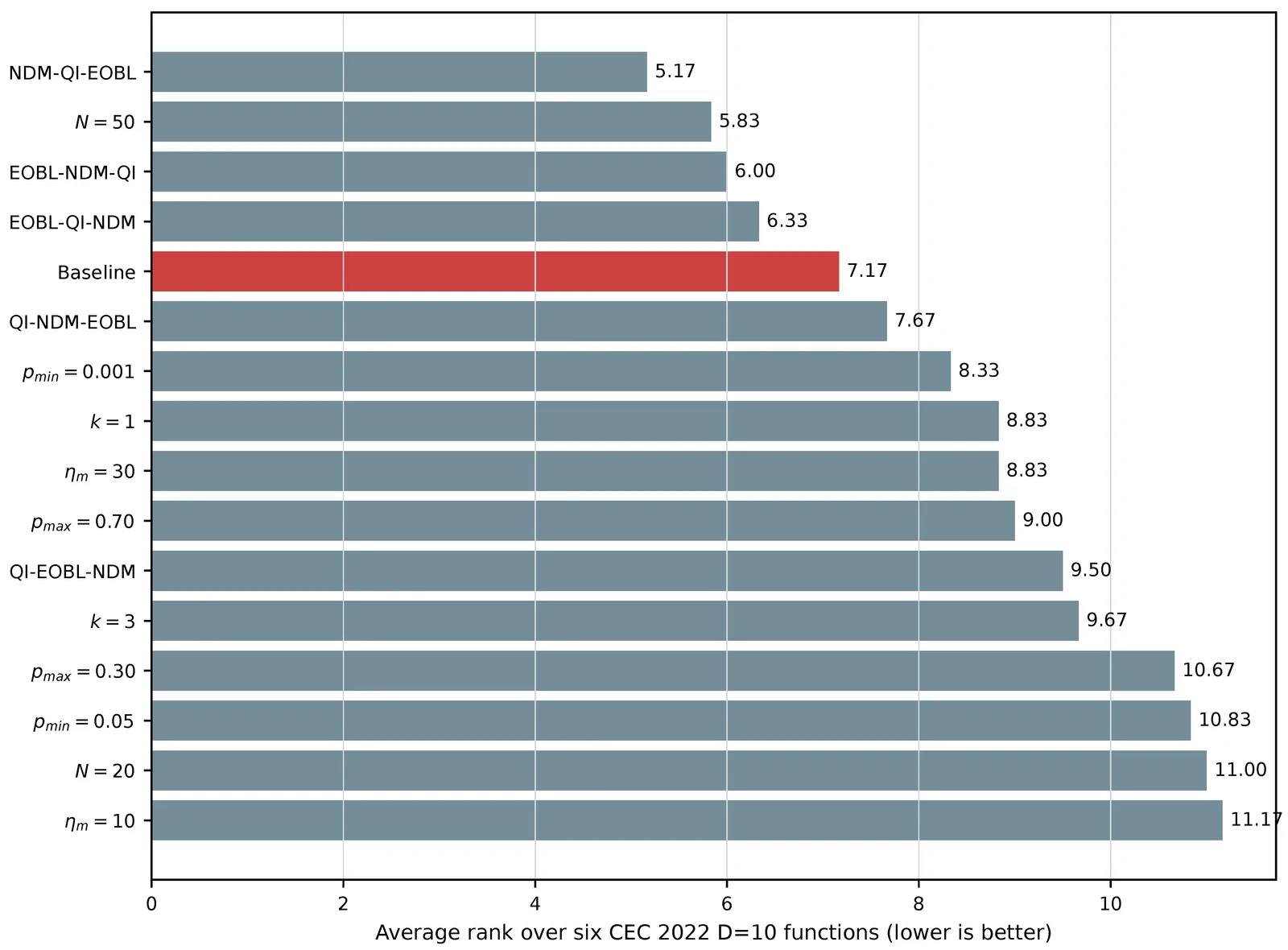
MSGSO ranks under one-at-a-time parameter variations; lower values indicate better performance. Red denotes the baseline MSGSO configuration, while blue-gray denotes the alternative parameter and operator-order profiles.

A one-at-a-time (OAT) parameter variation analysis was conducted on CEC 2022 functions F1, F4, F6, F8, F10, and F12 at D=10. Each profile used 10 matched seeds and 15,000 evaluations per run. The analysis varied $p_{\max}$, $p_{\min}$, *k*, $\eta_{m}$, population size, and the six possible operator orders, while the remaining settings were kept fixed. The purpose of this analysis is to examine the performance and ranking behavior of MSGSO under individual parameter and operator-order changes rather than to quantify global parameter influence. The 16 profiles produced 960 runs and 14,400,000 evaluations. Their average ranks are given in Table 9 and Figure 4.

The baseline configuration ranked 7.167, near the middle of the tested profiles. Moving QI ahead of EOBL produced the best rank (5.167), and increasing the population to N=50 produced the next best value (5.833). The weakest profiles used N=20 (11.000) or $\eta_{m}=10$ (11.167). Performance varies with operator order and population size within this test set. These observations were not used to replace the baseline parameters in the main comparison, which had been fixed before the parameter variation runs.

### 4.5. Convergence Analysis

**Figure 5:**
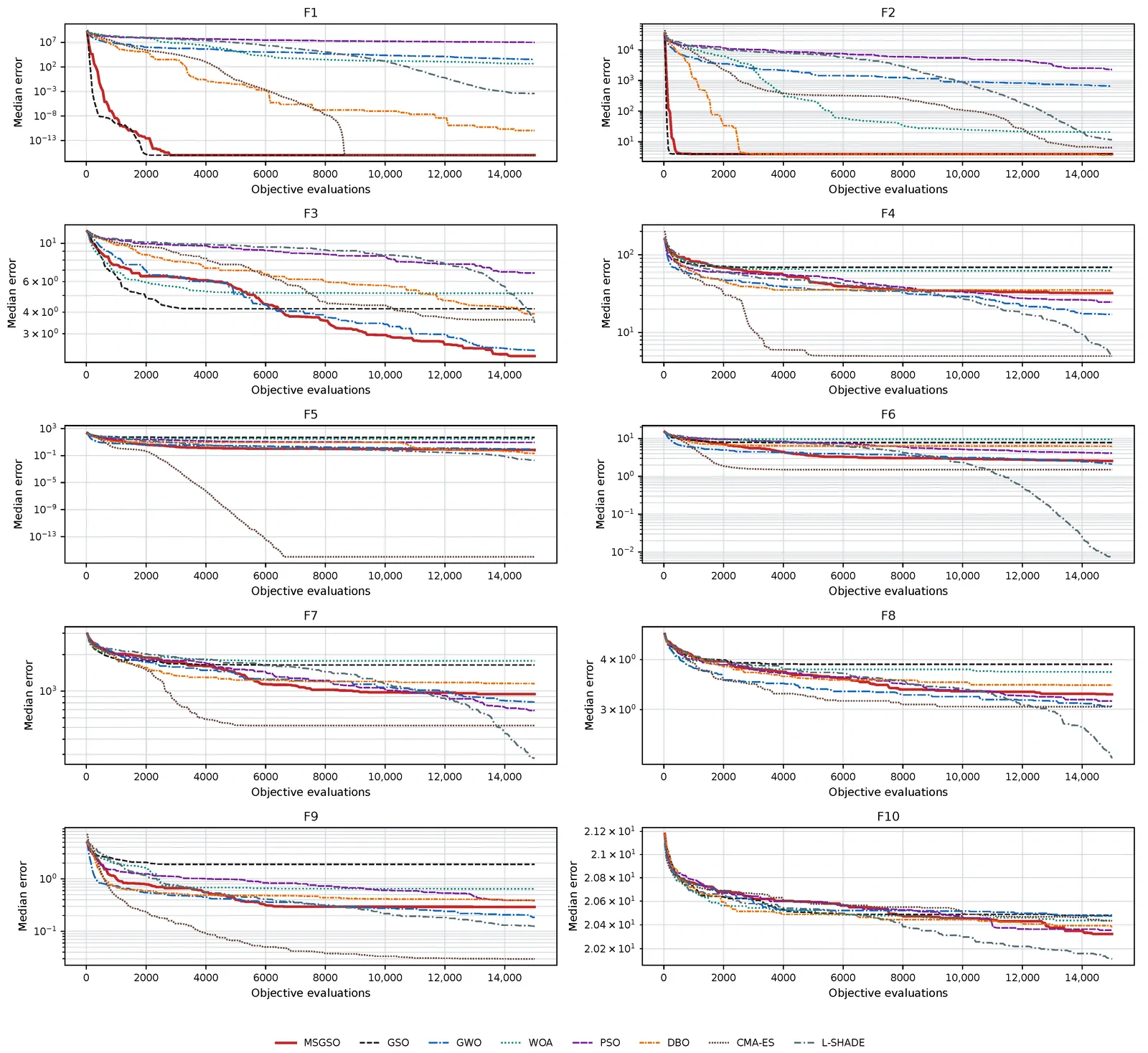
Evaluation-indexed convergence on all CEC 2019 functions. F1–F10 denote the ten CEC 2019 benchmark functions listed in Table 1.

**Figure 6:**
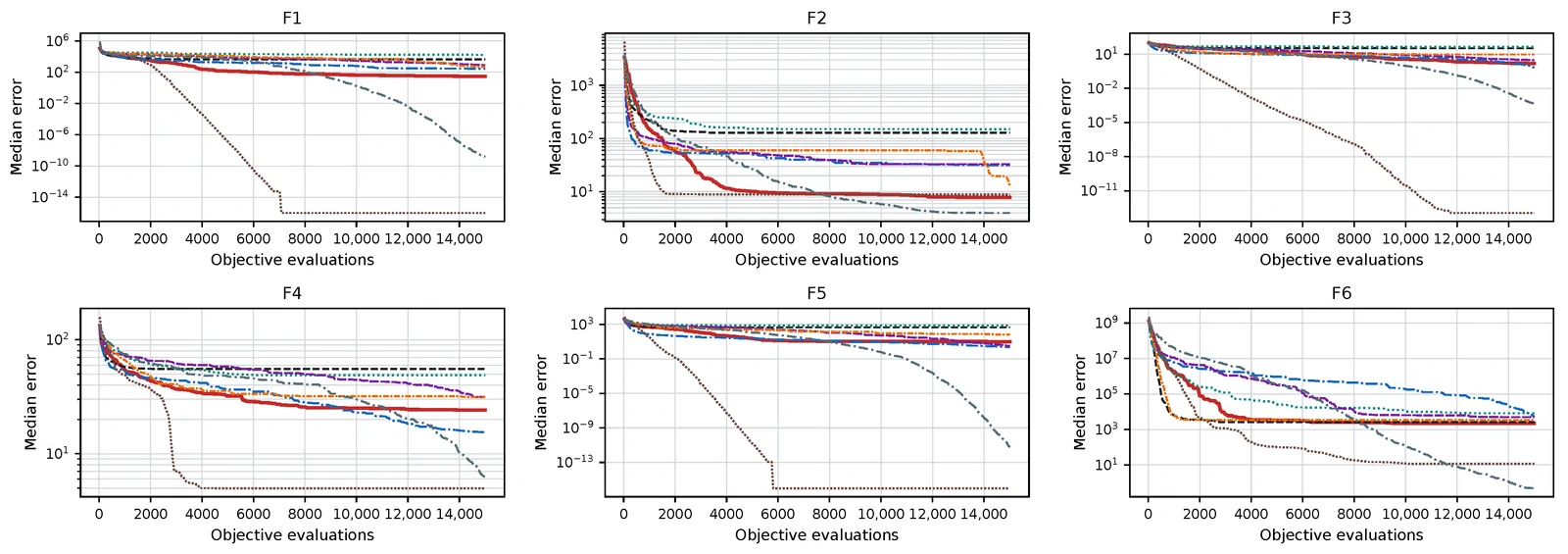

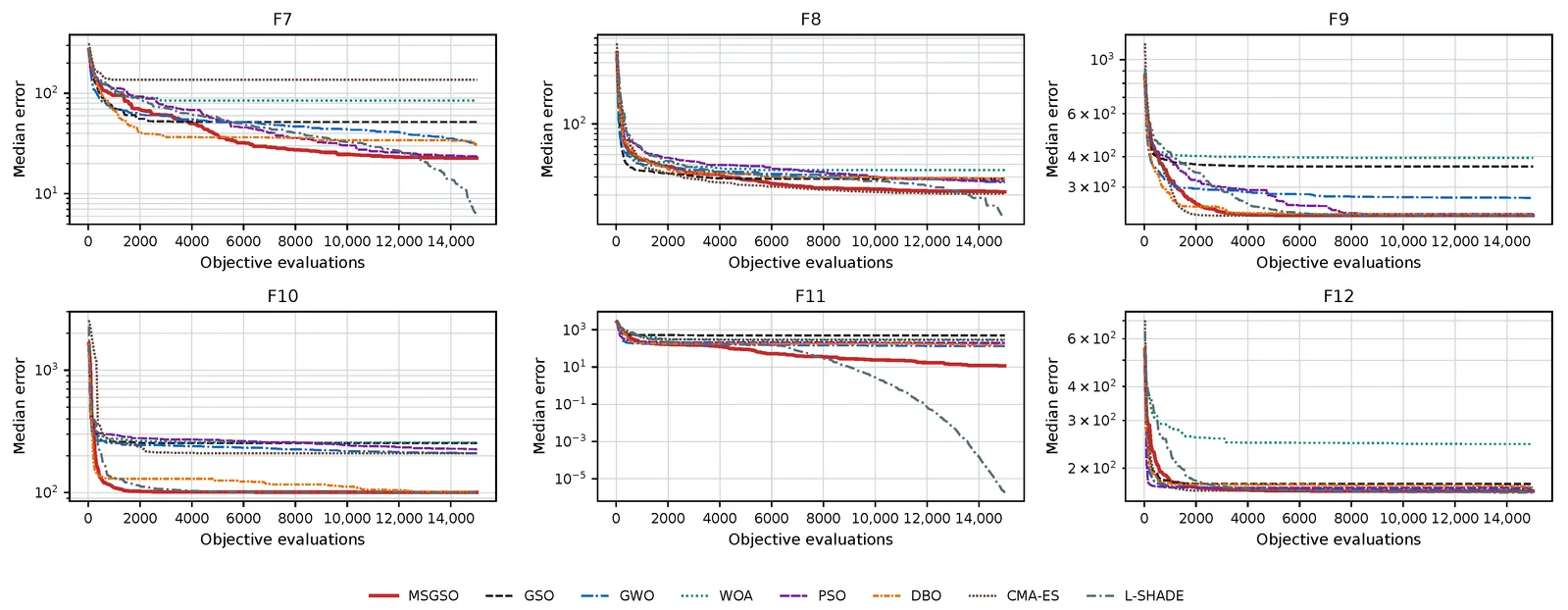
Evaluation-indexed convergence on all CEC 2022 functions at D=10. F1–F12 denote the twelve CEC 2022 benchmark functions listed in Table 2.

**Figure 7:**
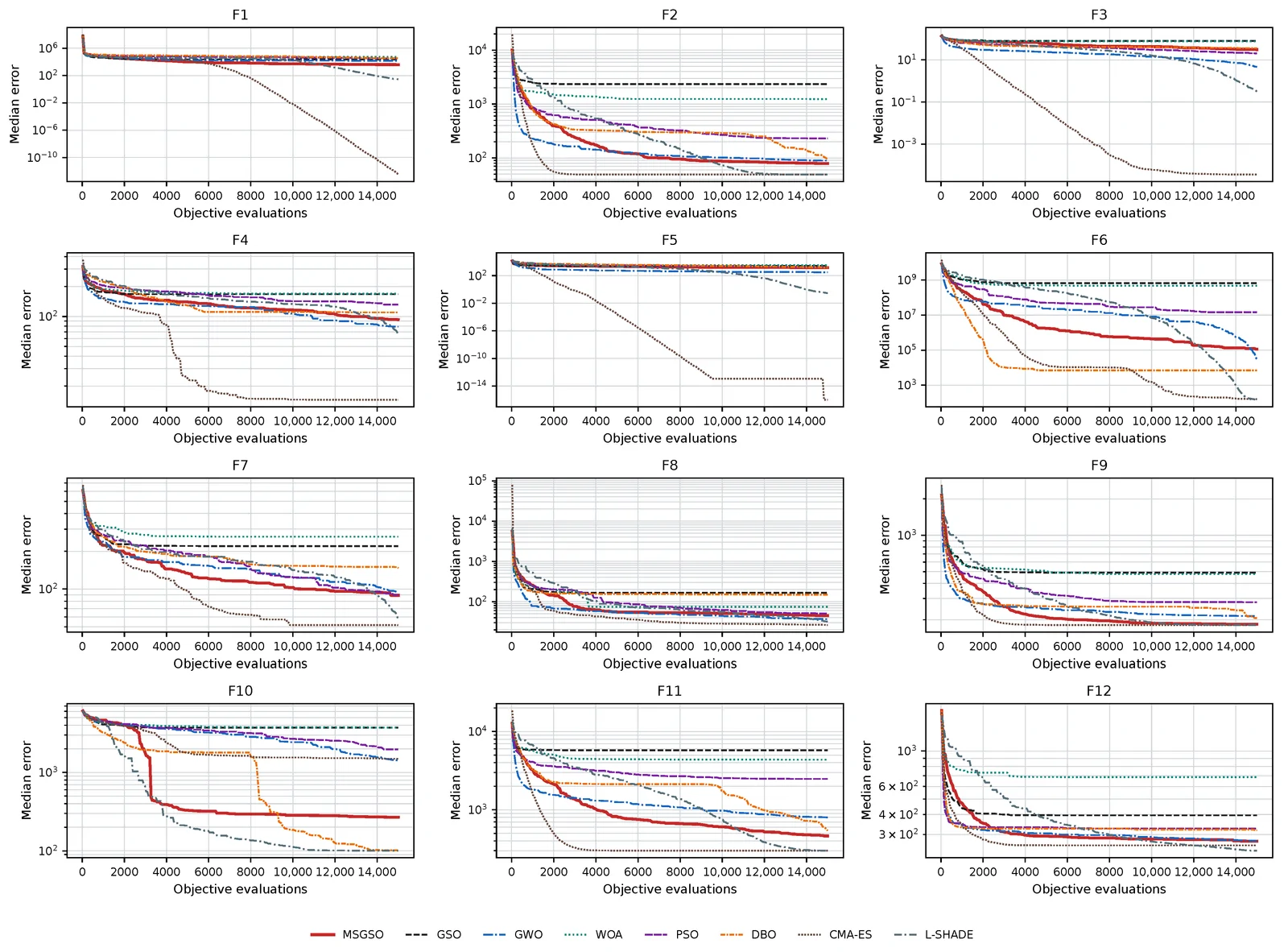
Evaluation-indexed convergence on all CEC 2022 functions at D=20. F1–F12 denote the twelve CEC 2022 benchmark functions listed in Table 2.

Figure 5, Figure 6 and Figure 7 plot the median best-so-far error over 30 runs against objective evaluations. The curves begin after the initial population evaluation and continue to the 15,000-evaluation limit. Axes, line styles, and legends are consistent across the three benchmark settings.

The trajectories support the final-rank analysis. On many functions, MSGSO continues to improve after GSO has reached a plateau. L-SHADE and CMA-ES, however, attain lower errors on several basic, hybrid, and composition functions within the same budget. Plotting against evaluations rather than iterations ensures that this comparison is not affected by the number of candidates generated in an algorithmic cycle.

## 5. Case Study: Three-Dimensional UAV Path Planning

### 5.1. Environment and Path Representation

The UAV study used the same MSGSO operators and parameter values as the benchmark experiments; no path-planning-specific retuning was performed. The objective combines path length and turning cost, while feasibility requires every segment to maintain the prescribed obstacle clearance. Consequently, a method can return a low penalized cost without reliably producing usable paths.

Three workspaces of size 30×30×15 were constructed from the cylindrical obstacle configurations in [13]. Their horizontal centers and physical radii are listed in Table 10 and reproduced exactly in the executable scenario file. Each cylinder spans the full altitude range. As a result, collision checks are two-dimensional, although altitude remains part of the path length and smoothness calculations.

The start and target positions are fixed at $P_{0}=\left(1,2,1\right)$ and $P_{11}=\left(26,29,1\right)$. Ten intermediate three-dimensional waypoints are optimized, so $P=\{P_{0},\ldots ,P_{11}\}$ and the decision dimension is D=30. Horizontal coordinates are restricted to [0,30] and altitude to [0,15]; all methods clip candidate waypoints to these limits.

### 5.2. Obstacle Avoidance and Safety Definition

For an obstacle *j* with horizontal center $o_{j}$ and radius $R_{j}$, the minimum planar distance between path segment *k* and the obstacle axis is

(14) $$d_{kj}=\min_{\lambda \in [0,1]}{∥\left(1-\lambda \right)P_{k,xy}+\lambda P_{k+1,xy}-o_{j}∥}_{2}.$$

A segment satisfies the clearance requirement when $d_{kj}\geq R_{j}+B$, with a fixed safety buffer of B=0.75. Before evaluation, any intermediate waypoint inside a safety disk is projected radially onto its boundary:

(15) $$P_{k,xy}\leftarrow o_{j}+\left(R_{j}+B\right)\frac{P_{k,xy}-o_{j}}{∥P_{k,xy}-o_{j}{∥}_{2}},$$

Two complete obstacle sweeps apply this deterministic repair. Moving the waypoints alone cannot guarantee that the connecting segments are safe, so the objective evaluates every segment independently. Define

(16) $$h_{kj}=\max\left(0,R_{j}-d_{kj}\right),\;s_{kj}=\max\left(0,R_{j}+B-d_{kj}\right),$$

The obstacle penalty implemented in the code is then

(17) $$C_{\mathrm{obs}}=\sum_{j}\sum_{k}\left[1000\;1\left(h_{kj}>0\right)+100\;1\left(s_{kj}>0\right)+10h_{kj}^{2}+s_{kj}^{2}\right].$$

The two indicator terms penalize physical intersections and safety-buffer violations separately; the squared terms additionally scale the penalty with the depth of each violation.

Let $v_{k}=P_{k+1}-P_{k}$. Path length and turning are measured by

(18) $$C_{\mathrm{len}}=\sum_{k}{∥v_{k}∥}_{2},\;C_{\mathrm{smooth}}=\sum_{k}\arccos\left(\frac{v_{k}^{T}v_{k+1}}{∥v_{k}{∥}_{2}{∥v_{k+1}∥}_{2}}\right).$$

$C_{\mathrm{alt}}$ is the sum of squared altitude-bound violations and is zero after clipping to [0,15]. The complete objective is

(19) $$F_{\mathrm{UAV}}=C_{\mathrm{len}}+50C_{\mathrm{obs}}+0.5C_{\mathrm{smooth}}+10C_{\mathrm{alt}}.$$

### 5.3. Experimental Results and Discussion

MSGSO, GSO, GWO, WOA, PSO, DBO, CPO [32], and SBOA [33] were evaluated in MATLAB R2026a Update 1 with N=30, 500 iterations, and seeds 1–30. The study produced 720 run files, each containing 500 complete iteration records. Every stored best path was evaluated again from its waypoint coordinates to verify segment clearances and constraint counts. The optimization histories were also checked for internal consistency.

The number of objective evaluations per run ranged from 13,288 to 45,530 because the algorithms generate different numbers of candidates in an iteration. Across all runs, the UAV study used 14,759,820 evaluations. The fixed iteration horizon compares the reliability of the final paths rather than evaluation efficiency. Convergence is also reported against the actual number of objective evaluations.

Feasible and infeasible paths were not pooled into a single cost ranking. Algorithms were ordered first by the proportion of feasible runs and, when those proportions were equal, by the median cost among feasible runs. Fisher’s exact test compared each feasibility proportion with that of MSGSO; the two-sided Mann–Whitney test compared conditional feasible costs. Both tests used α=0.05, and the reported *p*-values were not adjusted for multiple comparisons. Table 11 and Table 12 provide the descriptive statistics and pairwise tests, respectively.

In Scenario 1, MSGSO and GWO each produced 29 feasible paths from 30 runs. Their feasible-run medians were 47.005 and 48.159, respectively. Neither the cost difference (p=0.186) nor the difference in feasibility proportions (p=1.000) was significant. GSO, by comparison, returned only four feasible paths, and its feasibility rate was significantly lower than that of MSGSO ($p=1.88\times 10^{-11}$). Because so few GSO runs were feasible, its conditional cost comparison should be read cautiously.

MSGSO returned a feasible path in every run of Scenarios 2 and 3. In Scenario 2, it had the lowest conditional median cost (42.809). CPO, SBOA, DBO, and GWO also completed all 30 runs feasibly, and the MSGSO cost distribution did not differ significantly from those of CPO (p=0.935) or SBOA (p=0.491). In Scenario 3, MSGSO placed third under feasibility-first ranking, behind SBOA and CPO. Its conditional costs again showed no significant difference from either method (p=0.620 and p=0.888). Thus, MSGSO was reliable in these static environments, but it was not the leading method in every scenario.

**Figure 8:**
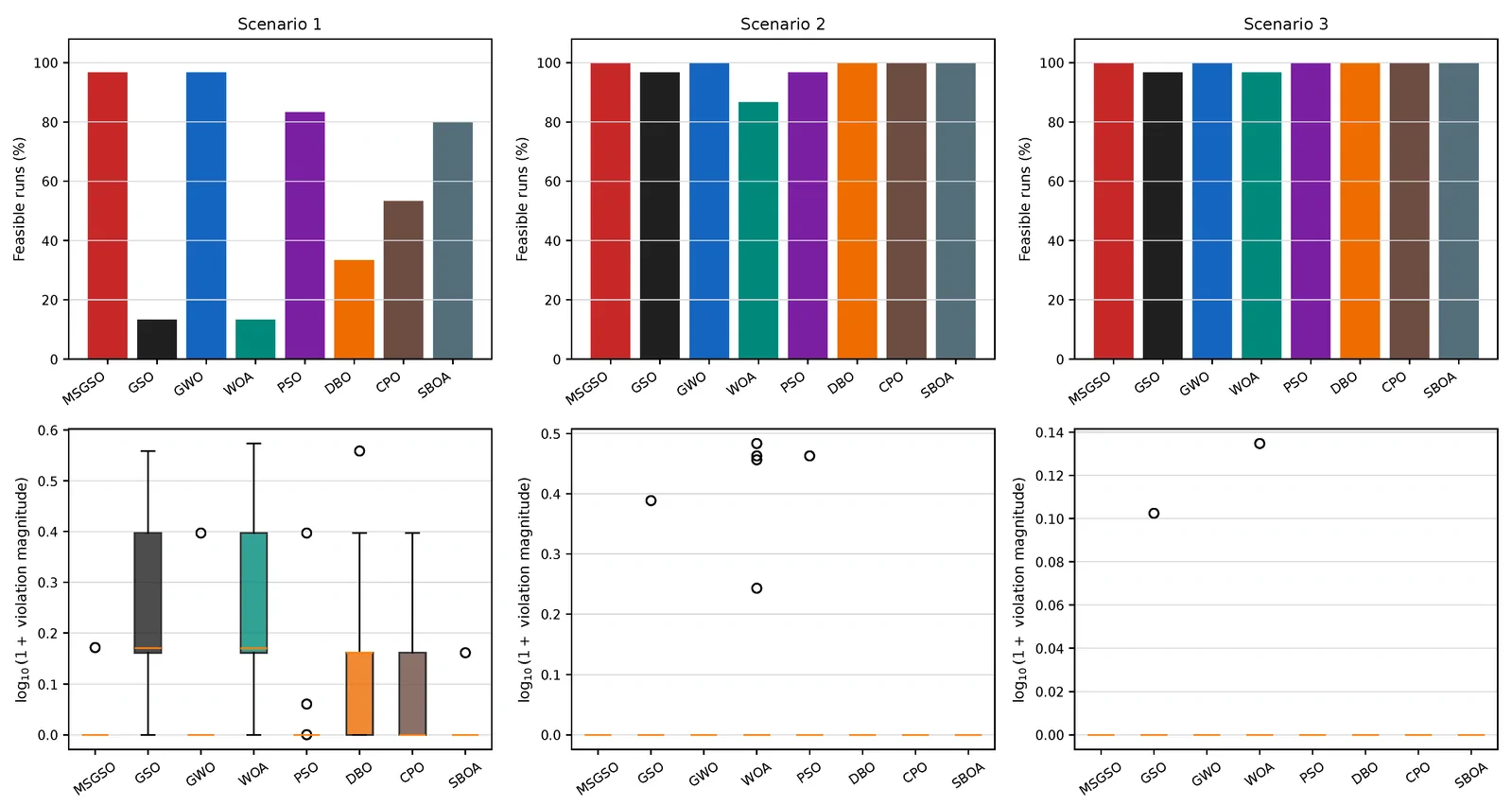
Run-level UAV feasibility rates and constraint-violation distributions after 500 iterations. Colors consistently distinguish the individual algorithms, and white circles denote outliers in the boxplots.

**Figure 9:**
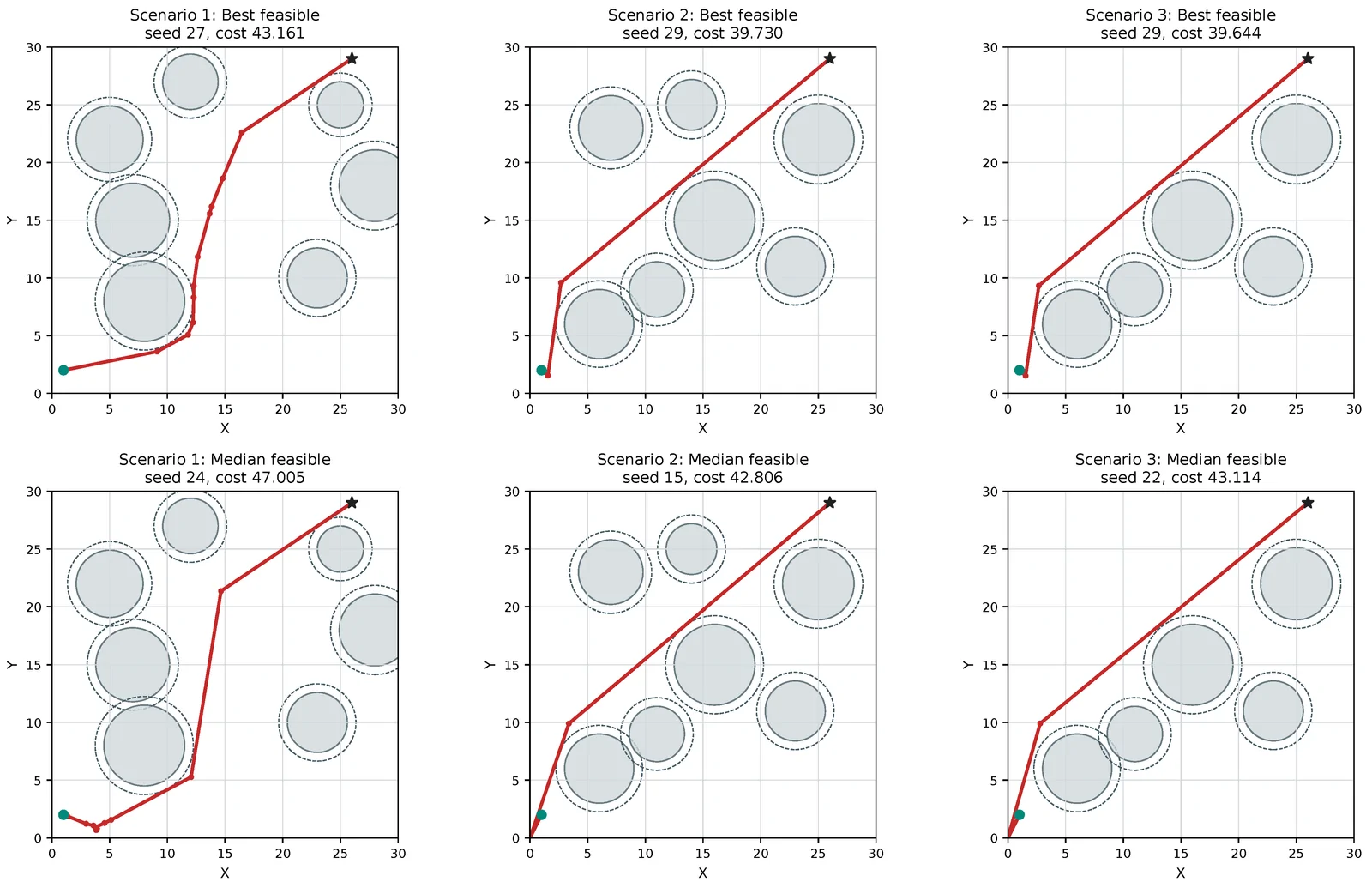
Best and representative median feasible MSGSO paths in the three scenarios. The red line denotes the UAV path, the green circle marks the start point, and the black star marks the goal point. Filled gray circles represent the physical obstacles, while dashed outer circles indicate the additional 0.75 safety buffer.

Figure 8 reports feasibility and violation magnitude separately. The methods differ most clearly in Scenario 1. In the other two scenarios, violation values are concentrated at zero because most runs are feasible. Figure 9 shows, for each scenario, both the lowest-cost feasible MSGSO path and the feasible path whose cost is nearest the conditional median. Presenting the median representative prevents the visual assessment from depending on a single exceptional run. Filled circles mark the physical obstacle radii, while dashed circles include the additional 0.75 safety buffer.

**Figure 10:**
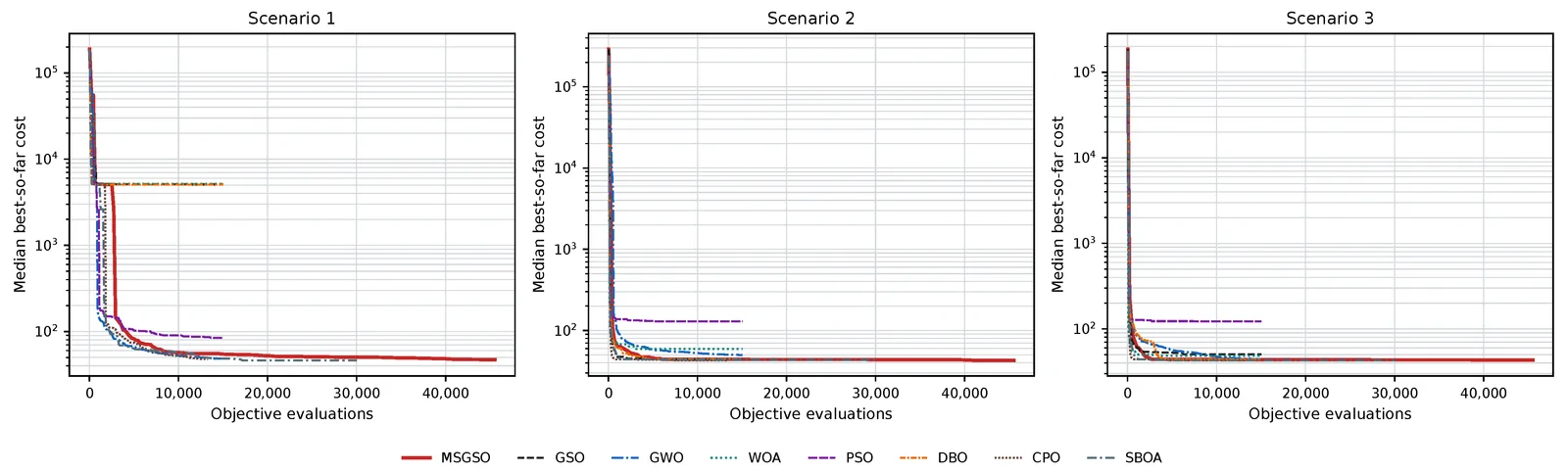
Median UAV best-so-far cost indexed by objective evaluations. Curves terminate at each algorithm’s 500-iteration evaluation count.

Figure 10 places the convergence histories on an objective-evaluation axis. MSGSO evaluates more candidates per iteration than GSO and the single-update comparators, which is visible in the different curve lengths. The UAV findings concern final-path reliability after 500 iterations; they do not establish an advantage in evaluation efficiency.

## 6. Conclusions and Future Work

MSGSO retains the original GSO chain update and weak-agent replacement, then applies NDM, EOBL, and QI in sequence. Its numerical performance was examined on CEC 2019 and CEC 2022 with 30 matched seeds and 15,000 objective evaluations for every algorithm–problem pair. The study combined an external comparison, complete-subset ablation, one-at-a-time parameter variation analysis, and evaluation-indexed convergence curves.

Under the shared budget, MSGSO’s W/T/L record against its parent was 8/2/0 on CEC 2019, 11/1/0 on CEC 2022 at D=10, and 12/0/0 at D=20. This improvement did not make MSGSO the leading numerical optimizer: it ranked third in each external comparison, behind L-SHADE and CMA-ES in different orders. MSGSO is competitive with the population-based methods considered here, but the results do not support a general claim of superiority over established numerical optimizers.

The ablation identifies the operators responsible for the improvement. NDM–GSO led the GSO variants on CEC 2019, and NDM–QI–GSO led them at both CEC 2022 dimensions. The full NDM–EOBL–QI sequence did not improve on these subsets under the same evaluation budget. NDM was the most consistent source of gain, and QI complemented it on CEC 2022. Operator order and population size also affected performance in the parameter variation study. These findings favor selective or adaptive use of the operators rather than assuming that all three should always be active.

The UAV study yielded a qualified result. MSGSO produced 29 feasible paths in 30 runs in Scenario 1 and feasible paths in every run of Scenarios 2 and 3. It led the feasibility-first ranking in the first two scenarios and placed third in the last. In Scenario 1, neither its feasibility rate nor its conditional path cost differed significantly from GWO. In Scenario 3, SBOA and CPO ranked ahead of MSGSO. These cases show that MSGSO can solve the tested static path-planning model reliably, but they do not extend to dynamic environments or establish evaluation efficiency beyond the stated 500-iteration horizon.

Further work should investigate adaptive operator activation, broader parameter variation studies across functions and dimensions, and constrained engineering problems evaluated under common objective-evaluation budgets. Future UAV experiments should consider dynamic and uncertain obstacles, energy and curvature constraints, multiobjective feasibility handling, and hardware-in-the-loop validation.

## Figures and Tables

**Table 1:** CEC 2019 benchmark functions.

ID	Function	*D*	Bounds
F1	Storn’s Chebyshev polynomial fitting	9	[−8192,8192]
F2	Inverse Hilbert matrix	16	[−16,384,16,384]
F3	Lennard–Jones minimum energy cluster	18	[−4,4]
F4	Shifted and rotated Rastrigin	10	[−100,100]
F5	Shifted and rotated Griewank	10	[−100,100]
F6	Shifted and rotated Weierstrass	10	[−100,100]
F7	Shifted and rotated modified Schwefel	10	[−100,100]
F8	Shifted and rotated expanded Schaffer F6	10	[−100,100]
F9	Shifted and rotated Happy Cat	10	[−100,100]
F10	Shifted and rotated Ackley	10	[−100,100]

**Table 2:** CEC 2022 benchmark functions.

ID	Function	Class	$f^{★}$
F1	Shifted and rotated Zakharov	Basic	300
F2	Shifted and rotated Rosenbrock	Basic	400
F3	Shifted and rotated expanded Schaffer F6	Basic	600
F4	Shifted and rotated non-continuous Rastrigin	Basic	800
F5	Shifted and rotated Levy	Basic	900
F6	Hybrid function 1	Hybrid	1800
F7	Hybrid function 2	Hybrid	2000
F8	Hybrid function 3	Hybrid	2200
F9	Composition function 1	Composition	2300
F10	Composition function 2	Composition	2400
F11	Composition function 3	Composition	2600
F12	Composition function 4	Composition	2700

**Table 3:** Implementation sources and fixed parameter settings.

Method	Source and Implementation	Fixed Settings
MSGSO/GSO	Authors’ MATLAB implementation; original GSO equations from [8]	N=30, RP=0.5; MSGSO: $p_{\max}=0.5$, $p_{\min}=0.01$, k=2, $\eta_{m}=20$; order: NDM–EOBL–QI
GWO	Formula implementation of [24]	N=30, a:2→0
WOA	Formula implementation of [25]	N=30, a:2→0, $a_{2}:-1\to -2$, b=1
PSO	Formula implementation of [26]	N=30, w:0.9→0.6, $c_{1}=c_{2}=2$
DBO	Formula implementation of [27]	N=30, producer fraction 0.2
CMA-ES	Hansen reference MATLAB code, v3.61.beta [28]	population 30, random bounded $x_{0}$, $\sigma_{0}=0.3\left(ub-lb\right)$; early-stop criteria disabled for budget matching
L-SHADE	Tanabe–Fukunaga release 1.0.1 [29]	$N_{init}=18D$, $N_{min}=4$, p=0.11, archive rate 1.4, memory size 5
All methods	MATLAB R2026a Update 1	30 matched seeds; 15,000 objective evaluations; clipping at global bounds

**Table 4:** Evaluation-matched ablation results with W/T/L counts based on paired Wilcoxon tests (α=0.05).

Setting	Variant	Average Rank	Win	Tie	Loss
CEC 2019	GSO	7.050	8	2	0
	NDM-GSO	2.350	0	8	2
	EOBL-GSO	5.050	7	3	0
	QI-GSO	6.250	7	2	1
	NDM-EOBL-GSO	3.050	1	8	1
	NDM-QI-GSO	2.650	0	7	3
	EOBL-QI-GSO	5.950	7	3	0
	MSGSO	3.650	Reference (N/A)		
CEC 2022, D=10	GSO	7.333	11	1	0
	NDM-GSO	2.750	0	10	2
	EOBL-GSO	5.750	11	1	0
	QI-GSO	6.750	11	0	1
	NDM-EOBL-GSO	3.667	1	11	0
	NDM-QI-GSO	1.750	0	10	2
	EOBL-QI-GSO	5.250	11	1	0
	MSGSO	2.750	Reference (N/A)		
CEC 2022, D=20	GSO	7.833	12	0	0
	NDM-GSO	2.417	0	9	3
	EOBL-GSO	5.500	12	0	0
	QI-GSO	7.083	12	0	0
	NDM-EOBL-GSO	2.833	0	12	0
	NDM-QI-GSO	2.333	1	7	4
	EOBL-QI-GSO	5.000	10	2	0
	MSGSO	3.000	Reference (N/A)		

**Table 5:** Evaluation-matched comparison with W/T/L counts based on paired Wilcoxon tests (α=0.05).

Setting	Algorithm	Average Rank	Win	Tie	Loss
CEC 2019	MSGSO	3.500	Reference (N/A)		
	GSO	6.400	8	2	0
	GWO	4.400	3	4	3
	WOA	6.900	10	0	0
	PSO	5.400	5	4	1
	DBO	4.700	5	4	1
	CMA-ES	2.400	2	3	5
	L-SHADE	2.300	3	0	7
CEC 2022, D=10	MSGSO	2.917	Reference (N/A)		
	GSO	6.750	11	1	0
	GWO	4.333	9	2	1
	WOA	7.667	12	0	0
	PSO	5.167	10	2	0
	DBO	4.917	10	2	0
	CMA-ES	2.833	2	4	6
	L-SHADE	1.417	0	0	12
CEC 2022, D=20	MSGSO	3.583	Reference (N/A)		
	GSO	7.417	12	0	0
	GWO	3.917	4	6	2
	WOA	7.333	12	0	0
	PSO	5.583	8	3	1
	DBO	4.917	7	4	1
	CMA-ES	1.500	1	0	11
	L-SHADE	1.750	0	0	12

**Table 6:** Median final objective values over 30 runs for CEC 2019 official dimensions; every run used 15,000 objective evaluations.

Algorithm	F1	F2	F3	F4	F5	F6	F7	F8	F9	F10
MSGSO	1.000	5.000	3.229	33.206	1.680	3.561	944.290	4.275	1.293	21.324
GSO	1.000	5.000	5.184	70.009	48.839	8.755	1640.689	4.890	2.891	21.479
GWO	3394.391	660.306	3.411	18.085	1.862	3.095	812.480	4.061	1.184	21.474
WOA	448.282	21.747	6.144	63.475	31.685	10.502	1774.474	4.725	1.647	21.437
PSO	$1.01\;\;\times \;\;10^{7}$	2270.306	7.734	25.668	9.819	5.092	692.643	4.151	1.387	21.355
DBO	1.000	4.656	4.912	35.777	1.215	7.184	1151.640	4.455	1.390	21.381
CMA-ES	1.000	7.434	4.609	5.975	1.000	2.500	518.965	4.050	1.030	21.435
L-SHADE	1.000	12.654	4.474	6.110	1.019	1.007	280.502	3.270	1.125	21.115

**Table 7:** Median final objective values over 30 runs for CEC 2022 at D=10; every run used 15,000 objective evaluations.

Algorithm	F1	F2	F3	F4	F5	F6	F7	F8	F9	F10	F11	F12
MSGSO	329.763	407.809	601.596	824.136	909.935	4006.142	2022.577	2221.449	2529.307	2500.696	2611.453	2864.630
GSO	4791.709	527.678	633.660	855.492	1349.515	4404.848	2051.867	2228.910	2664.619	2653.551	3096.318	2874.878
GWO	580.081	431.480	600.740	815.419	902.357	7074.125	2031.048	2226.559	2571.020	2609.735	2733.086	2866.065
WOA	$1.67\;\;\times \;\;10^{4}$	550.284	645.343	848.823	1613.356	9968.617	2084.647	2235.061	2695.531	2656.897	2878.735	2945.562
PSO	1089.280	432.968	602.966	831.340	903.130	6764.435	2023.541	2227.230	2532.929	2626.164	2796.533	2869.163
DBO	778.515	413.074	609.492	831.296	965.314	5214.454	2030.717	2228.964	2531.734	2501.343	2750.488	2869.550
CMA-ES	300.000	408.916	600.000	804.975	900.000	1811.653	2136.533	2220.458	2529.284	2609.396	2900.000	2865.033
L-SHADE	300.000	403.987	600.000	806.254	900.000	1800.484	2006.070	2212.506	2529.284	2500.297	2600.000	2862.700

**Table 8:** Median final objective values over 30 runs for CEC 2022 at D=20; every run used 15,000 objective evaluations.

Algorithm	F1	F2	F3	F4	F5	F6	F7	F8	F9	F10	F11	F12
MSGSO	4322.979	478.836	630.666	892.958	2382.713	$1.17\;\times \;10^{5}$	2089.045	2245.175	2483.904	2669.486	3062.901	2972.366
GSO	$2.39\;\;\times \;\;10^{4}$	2746.603	678.000	967.658	3499.654	$6.68\;\;\times \;\;10^{8}$	2220.015	2366.672	2789.813	6122.858	8363.898	3095.502
GWO	$1.51\;\;\times \;\;10^{4}$	489.137	604.645	878.823	1219.857	$3.20\;\;\times \;\;10^{4}$	2094.717	2236.837	2514.821	3824.799	3400.978	2973.312
WOA	$5.57\;\;\times \;\;10^{4}$	1641.053	674.875	969.857	4216.186	$4.75\;\;\times \;\;10^{8}$	2261.143	2274.254	2777.261	6108.569	6967.518	3386.147
PSO	$3.31\;\;\times \;\;10^{4}$	629.386	619.982	931.799	2535.730	$1.47\;\;\times \;\;10^{7}$	2089.949	2250.880	2579.772	4363.342	5079.229	3026.862
DBO	$3.55\;\;\times \;\;10^{4}$	496.702	635.961	910.045	2410.615	8812.228	2147.186	2348.783	2506.621	2501.867	3143.724	3018.146
CMA-ES	300.000	449.084	600.000	814.553	900.000	1957.694	2051.582	2226.163	2480.781	3891.386	2900.000	2956.215
L-SHADE	329.318	449.086	600.311	864.748	900.292	1945.584	2056.436	2232.047	2480.783	2500.687	2900.721	2937.383

**Table 9:** One-factor-at-a-time parameter and operator-order variation results on six CEC 2022 *D* = 10 functions.

Profile	Average Rank
NDM-QI-EOBL	5.167
N=50	5.833
EOBL-NDM-QI	6.000
EOBL-QI-NDM	6.333
Baseline	7.167
QI-NDM-EOBL	7.667
$p_{\min}=0.001$	8.333
$\eta_{m}=30$	8.833
k=1	8.833
$p_{\max}=0.70$	9.000
QI-EOBL-NDM	9.500
k=3	9.667
$p_{\max}=0.30$	10.667
$p_{\min}=0.05$	10.833
N=20	11.000
$\eta_{m}=10$	11.167

**Table 10:** Cylindrical obstacle parameters for the three UAV scenarios.

Scenario	Center *x*	Center *y*	Radius *R*
1	7.0	15.0	3.2
	23.0	10.0	2.6
	8.0	8.0	3.5
	25.0	25.0	2.0
	5.0	22.0	2.9
	12.0	27.0	2.4
	28.0	18.0	3.1
2	6.0	6.0	3.0
	11.0	9.0	2.4
	16.0	15.0	3.5
	7.0	23.0	2.8
	14.0	25.0	2.2
	23.0	11.0	2.6
	25.0	22.0	3.1
3	6.0	6.0	3.0
	11.0	9.0	2.4
	16.0	15.0	3.5
	23.0	11.0	2.6
	25.0	22.0	3.1

**Table 11:** Feasibility-first UAV results after 500 iterations and 30 runs. Cost statistics include feasible runs only.

Scenario	Algorithm	Feasible	Rate (%)	Feasible Median	Violation Median	Violation IQR	Rank
1	MSGSO	29/30	96.7	47.005	0.000	[0.000, 0.000]	1
	GWO	29/30	96.7	48.159	0.000	[0.000, 0.000]	2
	PSO	25/30	83.3	80.219	0.000	[0.000, 0.000]	3
	SBOA	24/30	80.0	45.584	0.000	[0.000, 0.000]	4
	CPO	16/30	53.3	45.962	0.000	[0.000, 0.449]	5
	DBO	10/30	33.3	65.533	0.449	[0.000, 0.449]	6
	GSO	4/30	13.3	78.119	0.479	[0.449, 1.495]	7
	WOA	4/30	13.3	133.397	0.478	[0.449, 1.495]	8
2	MSGSO	30/30	100.0	42.809	0.000	[0.000, 0.000]	1
	CPO	30/30	100.0	42.811	0.000	[0.000, 0.000]	2
	SBOA	30/30	100.0	43.395	0.000	[0.000, 0.000]	3
	DBO	30/30	100.0	43.979	0.000	[0.000, 0.000]	4
	GWO	30/30	100.0	49.974	0.000	[0.000, 0.000]	5
	GSO	29/30	96.7	42.680	0.000	[0.000, 0.000]	6
	PSO	29/30	96.7	127.495	0.000	[0.000, 0.000]	7
	WOA	26/30	86.7	50.945	0.000	[0.000, 0.000]	8
3	SBOA	30/30	100.0	42.806	0.000	[0.000, 0.000]	1
	CPO	30/30	100.0	42.811	0.000	[0.000, 0.000]	2
	MSGSO	30/30	100.0	43.063	0.000	[0.000, 0.000]	3
	DBO	30/30	100.0	43.979	0.000	[0.000, 0.000]	4
	GWO	30/30	100.0	44.467	0.000	[0.000, 0.000]	5
	PSO	30/30	100.0	122.860	0.000	[0.000, 0.000]	6
	WOA	29/30	96.7	47.995	0.000	[0.000, 0.000]	7
	GSO	29/30	96.7	49.307	0.000	[0.000, 0.000]	8

**Table 12:** Unadjusted two-sided MSGSO pairwise tests for UAV feasibility and feasible-run cost.

Scenario	Comparator	Fisher Exact *p*	Mann–Whitney *p*
1	CPO	$1.70\;\;\times \;\;10^{-4}$	0.045
	DBO	$2.29\;\;\times \;\;10^{-7}$	$5.96\;\;\times \;\;10^{-3}$
	GSO	$1.88\;\;\times \;\;10^{-11}$	$9.48\;\;\times \;\;10^{-3}$
	GWO	1.000	0.186
	PSO	0.195	$8.44\;\;\times \;\;10^{-9}$
	SBOA	0.103	0.272
	WOA	$1.88\;\;\times \;\;10^{-11}$	$4.89\;\;\times \;\;10^{-5}$
2	CPO	1.000	0.935
	DBO	1.000	0.020
	GSO	1.000	0.529
	GWO	1.000	$7.66\;\;\times \;\;10^{-5}$
	PSO	1.000	$4.46\;\;\times \;\;10^{-11}$
	SBOA	1.000	0.491
	WOA	0.112	$2.95\;\;\times \;\;10^{-6}$
3	CPO	1.000	0.888
	DBO	1.000	0.058
	GSO	1.000	0.020
	GWO	1.000	0.137
	PSO	1.000	$4.08\;\;\times \;\;10^{-11}$
	SBOA	1.000	0.620
	WOA	1.000	$3.16\;\;\times \;\;10^{-5}$

## Data Availability

The source code, fixed configurations, raw MATLAB run outputs, integrity-checking scripts, statistical analysis scripts, generated tables, and figure-generation files supporting the findings of this study are available from the corresponding author upon reasonable request.
